# Defining Socially-Based Spatial Boundaries in the Region of Peel, Ontario, Canada

**DOI:** 10.1186/1476-072X-10-38

**Published:** 2011-05-21

**Authors:** Adam Drackley, K Bruce Newbold, Christian Taylor

**Affiliations:** 1 School of Geography & Earth Sciences, McMaster University, 1280 Main St. West, Hamilton ON L8S 4K1, Canada; 2 McMaster Institute of Environment & Health (MIEH), McMaster University, 1280 Main St. West, Hamilton ON L8S 4K1, Canada

## Abstract

**Background:**

The purpose of the project was to delineate a series of contiguous neighbourhood-based "Data Zones" within the Region of Peel (Ontario) for the purpose of health data analysis and dissemination. Zones were to be built on Census Tracts (N = 205) and obey a series of requirements defined by the Region of Peel. This paper explores a method that combines statistical analysis with ground-truthing, consultation, and the use of a decision tree.

**Data:**

Census Tract data for Peel were derived from the 2006 Canadian Census Master file.

**Methods:**

Following correlation analysis to reduce the data set, Principal Component Analysis was applied to the data set to reduce the complexity and derive an index. The Getis-Ord Gi*statistic was then applied to look for statistically significant clusters of like Census Tracts. A detailed decision tree for the amalgamation of remaining zones and ground-truthing with Peel staff verified the resulting zones.

**Results:**

A total of 15 Data Zones that are similar with respect to socioeconomic and sociodemographic attributes and that met criteria defined by Peel were derived for the region.

**Conclusion:**

The approach used in this analysis, which was bolstered by a series of checks and balances throughout the process, gives statistical validity to the defined zones and resulted in a robust series of Data Zones for use by Peel Public Health. We conclude by offering insight into alternative uses of the methodology, and limitations.

## Background

Independent of individual characteristics, it is recognized that an individual's immediate environment possesses both material and social characteristics that are linked to health status as well as health-seeking behaviours [1-3]. That is, health reflects both individual characteristics, as well as the characteristics of the neighbourhood which constrains and enables individual health. For example, neighbourhoods may provide important information and support with regard to health practices and behaviours, but may also be associated with poor health in cases where crime is higher or the physical environment is poorer [4]. Concurrently, there is a common need for health status and related data to be represented at the 'neighbourhood' scale, whether it is for the provision of social welfare programs, planning, or health care delivery.

Geographers have long been concerned with defining neighbourhoods and places, and examples of techniques to define neighbourhoods abound in the academic literature [see, for example: 2, 3, 5-10]. Weden et al. [10], for example discuss the evolution and theoretical foundations, including links to public health issues, associated with neighbourhood classification, starting with the Chicago School. However, there are many approaches to defining zones, ranging from simple cases that are based on existing or historical neighbourhoods, school catchments zones, and communities, to more complex approaches including hierarchical clustering and scale-space approaches [see, for example, 11-13]. But even the so-called 'simple' cases can have fuzzy boundaries that are not agreed upon by residents and authorities alike, and new suburban communities may not self-identify as a cohesive neighbourhood, meaning that how areas are defined has been approached differentially based on the application. Most use various measures, such as poverty or educational attainment, that are derived from statistical organizations (such as Statistics Canada or the US Census Bureau), and represent a proxy for health outcomes. For example, the City of Toronto's 'Major and Minor' Health Planning Areas used the proportion of the population living in low income at the Census Tract level [2]. In Scotland, the identification of data zones was based on the Townsend Deprivation Index [9]. The City of Ottawa, Canada, analyzed physical and demographic characteristics of neighbourhoods through the so-called 'wombling technique' that analytically grouped areas based on statistical similarities, with the results providing an approximation of neighbourhoods [8,14]. They then went on to use a combination of ground-truthing, spatial analytical techniques, and GIS to define neighbourhoods. However, the wombling technique itself may be subject to validation inconsistencies based on the starting point of the analysis. Similarly, the use of simple, additive structures or the reliance on one particular population attribute to identify similar areas has also been criticized.

Despite attention and numerous papers on the topic, there is no one ideal (or recognized) way to define neighbourhoods and their spatial boundaries, and a lack of consensus remains as to the empirical definition of neighbourhoods. Often times, however, zones are constructed to reflect or identify differences in health across space [i.e., 1-3, 6-8]. But health is defined by more than just personal health and access to health care services. For example, the Determinants of Health framework [15,16] - which represents a synthesis of public health and social science literature and includes issues such as lifestyle options (i.e., drinking, smoking, physical activity), nutrition, housing, work, education, income, as well as mechanisms related to societal power, social identity, social status and control over life circumstances are influential in the distribution of health - suggests that these various place-based effects influence health at the neighbourhood scale [4,17]. Since they can be used to help contextualize and define neighbourhoods empirically, as opposed to more intuitive or theoretical conceptualizations [6], these place-based effects have formed the core of multiple papers on neighbourhood definition.

Multivariate techniques, geographic information systems (GIS), and spatial analytical (SA) techniques further enable understanding of neighbourhoods and their geography. For instance, GIS enables the visualization of neighbourhoods, while spatial analysis and cluster detection techniques such as the Getis-Ord Gi* statistic [18] provide a statistically robust way to identify areas that share statistically similar characteristics by identifying clusters of census tracts with values higher in magnitude than might be expected by random chance. If such statistical techniques are coupled with expert opinion and a clear decision process on boundary placement, approaches that use a mix of techniques may provide better area-based definitions. Ultimately, these neighbourhoods can be used to further understand health (or other) differences across space, and the relationship between place and health.

The question at hand is how to appropriately define aggregate neighbourhoods ('Data Zones') in the Region of Peel, Ontario. The project was initiated by Peel Public Health, who contacted the research team in mid-2009. The overall purpose of the project was to delineate a series of contiguous Data Zones within the Region for the purpose of health data dissemination. The use of the term 'Data Zones', as opposed to neighbourhoods, was preferred, since neighbourhoods typically have some degree of social identification associated with them and are frequently geographically smaller than the areas that would ultimately be identified in this project. The desired outcome, as requested by Peel Public Health, was to accomplish the following three goals:

• Develop a methodology for defining Data Zones within the Region of Peel while accounting for sociodemographic and socioeconomic effects;

• Use the Data Zones to describe selected health issues and outcomes across space;

• Analyze and report findings, such as the differences in health outcomes between spatial areas.

The resulting Data Zones are not intended to facilitate the delivery of services, but to identify relationships between inequalities in neighbourhoods and health disparities, with Peel Public Health using the zones as a communications vehicle; for reporting to people who have an interest in certain geographic areas; for planning purposes at the strategic level; and for following relevant trends over time.

The research team was therefore charged with developing a methodology to delineate internally homogenous Data Zones using geographic data with relevant software based on the 2006 Census, and using Census Tracts as the existing boundaries from which to build the zones. The purpose of this paper is therefore to illustrate a multivariate-structured technique [19] for the derivation of a series of Data Zones in the Region of Peel, Ontario. Following the selection of variables used to characterize and contextualize Census Tracts relative to health outcomes, GIS and spatial analysis techniques were used to map and construct Data Zones within the Region using the Getis-Ord Gi* statistic [18]. The Gi* statistic identifies 'hot-spots' or statistically significant clusters of similar Census Tracts, providing a statistically robust definition of neighbourhoods. The delineation of Data Zones is further facilitated by a structured decision tree approach, 'ground-truthing' with staff from Peel, and the overlay of existing neighbourhoods, road and other physical landforms to ensure appropriate representation and delineation of the zones. As such, the methodology to define zones is a heuristic approach, rather than an optimization method utilized in other studies, but one that provides a robust way to define zones.

## Data

**Figure 1:**
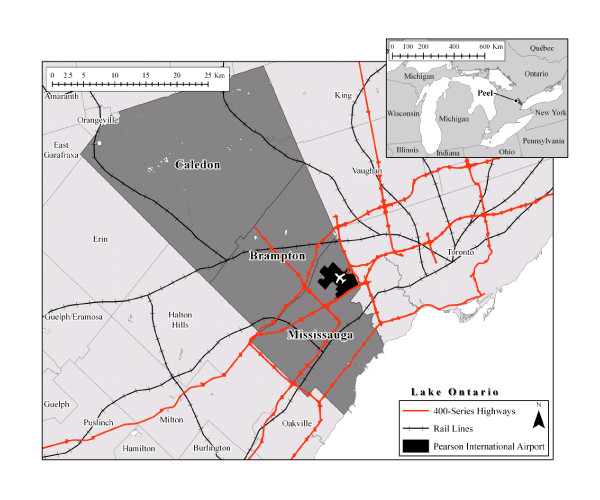
**Regional Municipality of Peel, Ontario**.

Lying to the west and northwest of the City of Toronto, the Region of Peel is part of the Greater Toronto Area (GTA) and the Toronto Census Metropolitan Area (CMA) (Figure 1). Peel is home to 1.1 million people (2006), making it the second largest municipality in Ontario, and includes the cities of Mississauga and Brampton and the towns of Caledon and Bolton. The Region can be roughly described as predominately urban and characterized by comparatively new large-tract suburban developments, although the northern portion (Caledon) of the region is predominately rural agricultural. As a regional government body, Peel is responsible for the services and infrastructure related to water delivery and wastewater treatment, policing, planning, and public health, amongst other services.

Given its proximity to Toronto, employment opportunities, and accessibility (home to Pearson International airport and served by seven '400' multi-lane, limited access highways), the region's population has grown rapidly. Between 2001 and 2006, the region grew by 17%, adding slightly more than 170,000 people to the population. Nearly 50% of Peel's population are born outside Canada (immigrants), with approximately 120,000 arriving between 2001 and 2006 alone. Large immigrant or visible minority groups (based on 2006 data) include South Asians (272,760), Filipino (42,900), Chinese (54,285), and Blacks (95,565). Other immigrant groups include South East Asian, West Asian, Latin American, Japanese, and Korean communities. Much of this new population is housed in new, low density suburban style development. Approximately 46% of the region's population report a non-English/non-French mother tongue. The median after tax income in Peel (2005) was greater than that of the overall province ($Cdn62,181 versus $Cdn52,117, respectively), and has a generally well-educated population, with 34% of the population aged 25 to 34 having a certificate, diploma, or degree [20].

Peel has a rich geography that is defined through multiple existing neighbourhoods or service planning boundaries, including older communities that continue to retain their identity and electoral boundaries. Existing planning and service delivery areas include Peel's 'Family of Schools' areas (used by the Peel District School Board), Forward Sortation Areas (used by Canada Post and as a basis for the Social Planning Council of Peel's 'Portraits of Peel'), Local Health Integration Networks, and Community Health Centre boundaries. Additionally, the Region is divided into a number of statistical zones, including Census Tracts (N = 205), which are small, relatively stable geographic areas that typically have a population of 2,500 to 8,000, and dissemination areas (400 -700 people), both of which are defined by Statistics Canada.

The purpose of this work was how to express these varied geographies and summarize the diverse sociodemographic and socioeconomic profiles of the Region. In the first instance, Census Tracts were used as the building blocks for the Data Zones given stated preferences by Peel Public Health and ease of data availability at this scale. In the second instance, and following a review of the relevant literature [i.e., 8] and requests by Peel Public Health, a set of variables were initially considered for inclusion in the analysis that the research team and Peel staff felt expressed Peel's diversity. Variables requested for consideration by Peel included: % with no knowledge of English or French; % aged 25+ years who completed less than high school; % recent immigrants; and % low-income population, all of which are used in comparable studies. All variables were derived from the 2006 Census and based on the 20% Master data file from Statistics Canada. In addition, the research team suggested a number of other variables linked to population health outcomes, including % unemployed, % visible minority, % labour force aged 15+, and average number of persons in a household. Other variables initially considered in the analysis included alternate measures of income (i.e., median income and after tax income).

From the initial list of 21 variables suspected by the Research Team to be likely indicators of health or socio-economic status, the number was reduced to 11 through correlation analysis (at the census tract scale) using SAS 9.2. In cases where two variables were highly correlated with each other (indicating that they are likely measuring the same outcome), one variable was removed from further consideration. While a specific correlation value above which variables were excluded was not used in the current analysis, the preference was to retain variables favoured by Peel and/or those supported by literature and that are linked to health outcomes. Variables retained in the analysis are defined in Table 1, and are consistent with those typically found in the literature and used for similar purposes. As such, we are explicitly acknowledging that no one, single variable could effectively summarize all neighbourhoods, with selected variables reflecting the literature on the determinants of health and relationships between environment and health outcomes [21]. For instance, poor housing conditions are commonly associated with poor health outcomes, and may alter the factors underlying health status both directly and indirectly, such as through the presence or absence of social support mechanisms [22-24]. Similarly, the amount spent on housing has been linked to health outcomes, with families that are forced to spend significantly more on housing potentially sacrificing other health-related needs, including food and health care [25]. Immigrants, and particularly visible minorities, have also been shown to have poorer health than the broader Canadian population, reflective of various barriers to care including language difficulties [26,27], knowledge of health care services, and socio-cultural roles [28]. Recent immigrant groups are also less likely to access health services in Canada than Canadian-born citizens [29,30], and are less aware of preventative health services [31]. Unemployment, low socioeconomic status, and low educational attainment are also commonly associated with poor health outcomes related to stress, inadequate knowledge of health care options and healthy lifestyles, and lack of income for health related activities [32-36].

**Table 1:** Variables included in Principal Components Analysis

Factor	Definition
Housing	% Renters
	% Owner households spending 30% or more of household income on major payments
	% Households in need of major repairs
Socioeconomic	% Aged 20+ with no High School
	% Unemployed (Prior to May 16^th^, 2006)
	% Low Income (Before tax, 2005)
Sociodemographic	% No Knowledge of English or French
	% Separated or Divorced
	% Widowed
	% Recent immigrants (Immigrated to Canada between 2001 and Census Day, May 16, 2006. (Census, 2006)
	% Lone Female Parent Family

Note: All variables derived from the 2006 Census.

## Methods

In defining Data Zones within the Region, Peel Public Health requested that the following issues be considered:

• Approximately 12-14 Data Zones were to be defined, with populations of approximately 80,000 to 100,000. The exception to this request was in the northern portion of the Region of Peel (the community of Caledon), which is predominantly rural and therefore has a smaller population density. Peel reserved the right to redefine the population threshold for zones following the initial analysis;

• Data zones were to be contiguous, follow Census Tract boundaries, and avoid cases where zones were encircled by other zones;

• Data zones were to follow boundaries that correspond to areas of interest for other purposes;

• Data zones were to focus more on the composition of the local population when defining neighbourhood boundaries, rather than neighbourhood context [i.e., 13, 31, 32];

• Where plausible, it was requested that the Data Zones respect natural and human-made boundaries, such as rivers and highways. Several such barriers exist within the Region of Peel, including rail lines, the Credit River which extends northwest through Mississauga, and limited-access highways including highways 401, 403, 407, 410, and the Queen Elizabeth Way (QEW) which dissect the region. In most cases, census tracts already follow these boundaries. In cases where they do not, census tracts must still form the boundary.

In practice, it was not possible to satisfy all these criteria, and compromise was necessary. Most commonly (and as noted below), population constraints were waived in consultation with Peel staff given future growth anticipated growth trends.

Differences in perceptions and definitions imply that neighbourhoods mean different things to different people (see Luginaah et al. [6] for a review). Although there is disagreement in the literature concerning the best way to capture the concept of a neighbourhood, Census Tracts provide one option. At the same time, the use of Census Tracts have been frequently been criticized because their statistically defined areas impose boundaries may not necessarily be related to other social processes or perceptions of what a neighbourhood includes, reducing the power of a neighbourhood as a meaningful concept [37-40]. On the other hand, other studies argue that Census Tracts are good proxies of neighbourhoods [1,3] as compared to socially constructed areas, which are often loosely defined and lack the ability to link to other statistical data. Indeed, the comparison of several neighbourhood units of analysis suggests that Census Tracts are good proxies for natural neighbourhood boundaries in studies of neighbourhood effects on health [3]. Moreover, defining neighbourhoods by using Census Tracts (or groups of Census Tracts) offer a number of advantages, including direct linkage to statistical measures provided by Statistics Canada.

Following the initial selection of the variables, principal component analysis (PCA) with a varimax orthogonal rotation was used to summarize variables and build indices, a practice commonly used to consolidate information along main dimensions and that has been widely used in defining zones similar to the aims of this work [i.e., 8, 41, 42, 43]. While other zoning exercises have constructed an index based directly on the weighted variables, indices constructed in this way may be misleading by missing inter-relationships between variables, and/or fail to account for a more complete set of potential indicators. The central idea of PCA is to reduce the dimensionality of a data set which consists of a large number of interrelated variables, while retaining as much as possible the variations present in the data set [42], allowing the determination of which tracts could be combined to form relatively homogenous areas. PCA allows for the extraction of components that reflect the pattern of the inter-correlations of the variables, while searching for commonalities. Only factors that contributed greater than 10% of the variation would be retained for further analysis.

While PCA assists with the identification of the sources of variation, it does not help in understanding the spatial patterning of the components. Following PCA, therefore, the next step was to create the boundaries for the zones based on the PCA scores assigned to each Census Tract for each factor created by PCA. For this purpose, a Getis-Ord Gi* hot-spot analysis [18] was run on the resulting sets of Factor Scores. The statistic works by looking at each tract within the context of neighbouring tracts: if a tract's value is high (low), and the values for the neighbouring tracts are also high (low), it is a part of a so-called 'hot spot'. For each PCA factor, the Gi* statistic identifies the association between a Census Tract and its neighbours up to a specified distance, or in terms of nearest neighbours where the CT shares a boundary. The Gi* statistic is well-suited to identify the existence of pockets or clusters of areas (tracts) with values higher in magnitude than might be expected by random chance and their statistical significance; to assess assumptions of stationarity (i.e., that spatial relationships are the same at all places in the study area); and to determine distances beyond which no discernible spatial association exists [18]. Importantly, the Gi* statistic identifies clusters that can be used to statistically delineate zones. The output of the Gi* function is a z-score for each feature, with the z-score representing the statistical significance of clustering for a specified distance, and the higher (or lower) the z-score, the stronger the association. A z-score near zero indicates no apparent concentration.

## Results

Two major factors emerged from the Principal Components Analysis (Table 2), explaining approximately 65% of the variance and which are often associated with poor health outcomes within the determinants of health literature. The first principal component, which explained 45% of the variance, is labelled as '*low socioeconomic status*' and includes the variables indicating a high recent immigrant population, no knowledge of either English or French, percent unemployed, no high school, and low income. The second component, which explained an additional 20% of the variance, was labelled as '*single renter'*. The variables that loaded into this component included Separated/Divorced Single Parent Families with high rates of Rental Housing tenure, need for Major Repairs, and Low Income before Tax status. The percentage variance explained by a third component (9.5%) was marginally less than the threshold typically used with PCA [42]. In addition, the variables that loaded highly into the third principal component (widowers and individuals without high school education), did not appear to be indicative of any particular social group, and were already included in component 1 (education) or component 2 (widower status). As such, it was not considered further in the current analysis.

**Table 2:** Final Component Eigenvalues

Eigenvalues (CORR)				
**Component**	**Eigenvalue**	**Difference**	**Proportion**	**Cumulative**

**1**	**4.972620**	**2.767540**	**0.4521**	**0.4521**
**2**	**2.205080**	**1.164828**	**0.2005**	**0.6525**
3	1.040252	0.233820	0.0946	0.7471
4	0.806432	0.145497	0.0733	0.8204
5	0.660934	0.201995	0.0601	0.8805
6	0.458939	0.176109	0.0417	0.9222
7	0.282830	0.069393	0.0257	0.9479
8	0.213437	0.062486	0.0194	0.9673
9	0.150951	0.020085	0.0137	0.9810
10	0.130866	0.053206	0.0119	0.9929
11	0.077660	---	0.0071	1.0000

Two methods of extraction for the resulting factors were tested. In the first method, following work done by Primpas et al. [43], a weighted index was created for the highest-scoring rotation correlations in each factor, rescaled so as to sum to a value of 1. These final rotation correlations are highlighted in Table 3. Factor scores were generated for each Census Tract by multiplying each census variable by its respective rescaled rotation correlation. The resulting summed value of these variables represents a score out of a possible 100 for which that particular Census Tract scores. The second method of extraction was equally as accurate (i.e., produced the same results) in terms of the final results. Upon performing a rotation, SAS outputs a value for each record representing how strongly each Census Tract scores in each of the rotated principal components. While both approaches yield similar visual results, with overlays of the two component values defining similar areas, the second method was utilized.

**Table 3:** Varimax Rotated Variable Rotation Correlations

Rotation Correlations (Structure)		
**Variable**	**RT1_4**	**RT2_4**

% Separated or Divorced	0.070103	**0.910449**
% Widowed	0.082087	**0.487650**
% Renters	0.478132	**0.766992**
% Households in need of major repairs	0.015941	**0.752548**
% No English or French	**0.843414**	-0.327763
% Unemployed	**0.743667**	0.222037
% No High School	**0.467324**	0.207493
% Low Income (Before tax, 2005)	**0.814993**	**0.448588**
% owners spending 30% or more	**0.836553**	0.204874
% Recent Immigrant	**0.878143**	0.131458
% Single Mothers	0.291777	**0.743309**

**Figure 2:**
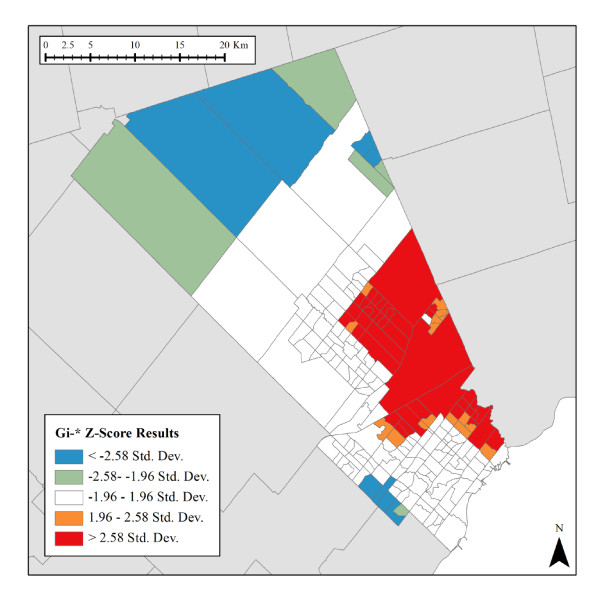
Low Socioeconomic Status Index (Gi* *p *< 0.05 clusters).

**Figure 3:**
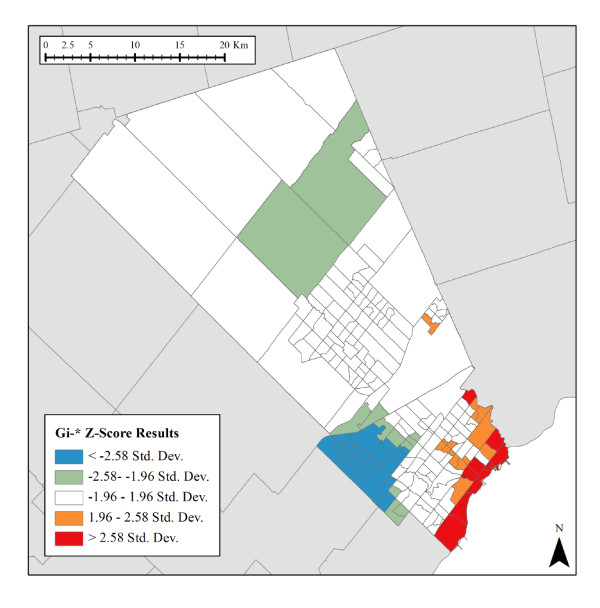
Single Renter Status Index (Gi* *p *< 0.05 clusters).

The two PCA factors were used as input to the Gi* analysis (Figures 2 and 3). A Manhattan Distance measurement was applied to reflect the predominately urbanized nature of region, and a Fixed Distance Band using the default distance of approximately 11 kilometres was ultimately applied. Only areas with a *p *value smaller than 0.05 (95% confidence) for either components were retained. In Figure 2, statistically significant spatial clustering of high Low Socioeconomic Status index values were found along the eastern extent of Peel, while the north and south contain statistically significant spatial clustering of low Low Socioeconomic Status index values. In Figure 3, statistically significant spatial clustering of Single Renter index values were found along the southeast waterfront and in the southwest and northwest areas of Peel. By analyzing patterns of discreteness and overlap between the two indices, Data Zones could be delineated based on these clusters.

Once the Gi* was computed and mapped for both PCA components, Data Zones could be delineated. As a first step, groups of Census Tracts that were statistically significant for either of the mapped components (low socioeconomic status and single renter) became the building blocks for a Data Zone. That is, there is statistical robustness based on the Gi* statistic for grouping these zones based on their similarity.

**Figure 4:**
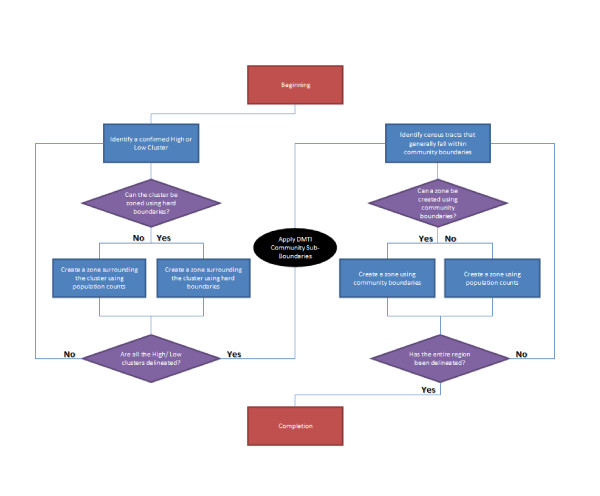
**Data Zones decision tree**.

While the Getis-Ord Gi* analysis allows the determination of 'hot-spots', portions of the region remained un-identified, including much of central Peel. That is, the Gi* analysis did not find statistically significant clusters for either factor in much of the central portion of the region. To derive Data Zones in these areas, a decision tree was created to allow for a reliable, repeatable delineation process that avoids personal subjectivity. The resulting decision tree (Figure 4) explains how the process of delineation followed a set of decisions based upon clustered factor scores, population counts, and community inclusiveness.

The general logic of the decision tree was based on two general streams. In cases where the Gi* analysis identified hot-spots, these clusters were compared with known hard boundaries such as roads or other features, and checked to ensure that they met other criteria such as population size. For the remaining portions of the Region that needed to be defined (i.e., those areas that were not defined as clusters by the Gi* statistic), we first turned to the DMTI Neighbourhood and Community Boundaries file [44], a "continually updated" set of neighbourhood boundaries as determined by "amalgamating and integrating information from municipal data sources" [45]. These DMTI-based boundaries were over-laid with the initial zones, allowing Data Zone boundaries to be initially constructed based on known neighbourhoods, while referencing population counts for each potential zone and ensuring that the constructed zones remained contiguous.

Once the initial set of contiguous zones was generated, a physical approach was used to refine zonal boundaries through two 'ground-truthing' methods. First, we referenced known boundaries, including physical features such as highways and streams, to determine if more 'natural' boundaries separating zones might be warranted, echoing Pickett and Pearl's [46] call for meaningful neighbourhoods that are based on natural boundaries. In this case, we assumed that such barriers differentiate areas through the physical division of space, such as separating neighbourhoods so that there is reduced interaction, or by separating places with different socioeconomic and sociodemographic profiles. Consequently, natural boundaries serve both functional purposes such as transport or recreation, as well as creating barriers between different groups [e.g., 47, 48]. Roads and highways were obtained from DMTI's Route Logistics file (2008), which contains highways and roads for all of Canada, albeit clipped to the boundaries of the Region of Peel [49]. Visible land features were obtained from the Satellite Streetview Orthophoto dataset created by the 60cm resolution Quickbird Satellite and released by DMTI Spatial [50]. The result is a spatial file with the different overlays (zones, neighbourhoods, roads, physical landforms), along with the zones delineated by the Gi*statistic, neighbourhood boundaries, and other "hard boundaries" (i.e., transportation) in Peel. Comparison of these boundaries identified any anomalies through consideration of both land features and physical boundaries. Throughout, total population counts for each potential zone were verified. The resulting 'shape' of the derived Data Zone was not an issue in the analysis owing to the imposition of the various constraints - statistical significance from the Gi* statistic, number of derived zones, known boundaries such as roads or physical features, and population counts - meant that any attempt to constrain the shape of the Data Zones was less meaningful. A total of 13 zones were identified at this stage of the analysis.

Second, we presented the results to Peel Public Health for their expert input on the defined boundaries. Peel staff, including GIS technicians, planners, and public health officials participated in two round-table discussions where interim results were presented. Through their more detailed knowledge of current and future population trends, socioeconomic profiles, and development within the region, participants critically analyzed the methodology and outcomes, and commented on potential anomalies or disagreements with the resulting divisions. These exercises resulted in the division of Caledon to create Data Zone 13 (West Brampton) and 15 (Bolton) at the request of Peel staff. In the first instance (zone 13), Peel's Official Plan notes the short-term housing and commercial development of the West Brampton area, with rapid population growth expected within a five-year window. Although the area was still largely rural (as of 2010) and therefore more similar to Caledon, the imminent population growth and development meant that Peel staff felt it was more suitable to present it as a separate zone rather than amalgamate with Caledon as suggested by the statistical analysis, enabling future flexibility with the zones. In the second case, the community of Bolton (Data Zone 15) was separated from the northeast portion of Brampton, again reflecting the uniqueness of the Bolton area (relative to the rural areas immediately around Bolton), and the potential for substantial short-term population growth, even though its 2006 population count (22,719) also falls below the threshold originally suggested for the definition of the zones. While counter to the initial constraints (namely that population thresholds for the two new Data Zones were less than the minimum size initially requested by Peel, meaning the population of the zones was not equitably distributed across each zone) and the clustering results, Peel staff felt that these modifications better provided for the future growth of Peel's population and more consistent zones over the longer-term. In addition to consultation with Peel staff, Peel also used the final derived Data Zones to produce maps of various health outcomes as an internal check of their validity.

**Figure 5:**
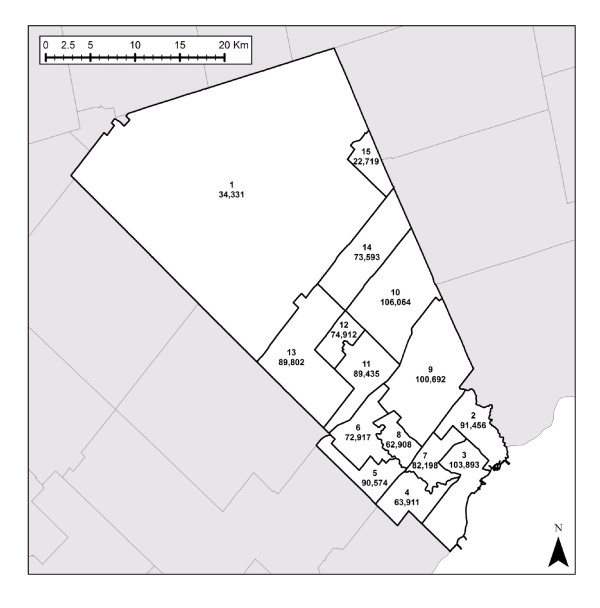
**Final Data Zones with Census Tract Population Counts**.

The result of the analysis, 'ground-truthing' and expert input exercises resulted in the set of Data Zones shown in Figure 5. Populations ranged from 22,719 (Bolton) to 106,064 (East Brampton) with boundaries that respect the various natural and physical delimiters in the region.

## Conclusion

Through a series of mixed methods, a set of Data Zones were delineated for the Region of Peel, Ontario, based on existing Census Tracts. It is hoped that as further census and health outcome data becomes available, and given that Peel's population continues to grow and become more diverse, the delineated zones can be verified and refined for future analyses.

The approach used in this paper is flexible and bolstered by a series of checks and balances throughout the process, including the use of statistically defined clusters of like Census Tracts through the use of the Gi* statistic, giving statistical validity to the defined zones. In addition, the use of a formal 'decision tree' to assist in the determination of zones, along with the recognition of local community boundaries, physical land features such as major roads or landscapes, and the knowledge of local health experts, resulted in a robust set of Data Zones for use by Public Health in the Region of Peel. Consequently, the methodology to define zones illustrated in this paper draws upon a number of inputs, with the end result a more robust and meaningful set of zones.

The methodology has a number of advantages, enabling it to be applied elsewhere and in different contexts. First, the method can be adjusted based on the desired output such as size/number of zones, their constituent building blocks, or even the inclusion (exclusion) of the initial statistical steps such as PCA. For example, the PCA analysis could be removed as a step. Instead, input for the Gi* analysis could, for example, be based on other existing inputs (i.e., an individual variable such as low income status) or indices, such as the UK's Townsend Index of Deprivation [9,51]. Following the Gi* analysis, which would identify clusters of like areas based on these alternate inputs, statistically similar zones could once again be identified through the use of the decision tree, consultation, and expert opinion. Second, the approach is applicable to both research and practical applications such as health surveillance. Third, the approach can be scaled up or down to other geographical contexts. Fourth, the consultative process and use of ancillary data removes concerns that the zones are only representative of the statistical process and the building blocks (Census Tracts) that underlie the zones. In essence, the proposed methodology increased participation in the analysis, and ultimately improved the definition of the resulting Data Zones to reflect local knowledge.

At the same time, the practice of using aggregated spatial data as the basis for creating larger areal units is a technique associated with potential errors, biases, and oversights - regardless of the context or application. First, the creation of socially-based spatial aggregations can be used to misrepresent those living within an area, either intentionally as in gerrymandering political districts to subdivide sizable voting populations, or unintentionally through irresponsible analysis. Caution must therefore be exercised in the use of expert opinion. Consequently, the decision tree is an important component of the work, providing a platform from which to evaluate changes to the set of zones.

Second, although Census Tracts were requested by Peel Public Health to be the building blocks for the analysis, there are reliability issues with using such a large spatial area as the building block for even larger Data Zones. It is recognized within spatial science that as an aggregated area increases in size, the recognized variance of the characteristics of the population within the area declines [52]. By generalizing the characteristics of a population with some kind of areal unit, potentially important variances within the defined zones are hidden. By using Census Tracts as opposed to smaller dissemination areas (for which the same census information is available), important variations in the population composition of the Region may be over-looked. The modifiable areal unit problem typifies this [52,53], reminding researchers that "the areal units (zonal objects) used in many geographical studies are arbitrary, modifiable, and subject to the whims and fancies of whoever is doing, or did, the aggregating" [53, p. 102]. Because of this, whenever attempting to subdivide an area based on the assumed similarities of those living there, care must be taken to ensure that the generalized areas most accurately represent the people living within their borders, maximizing the differences between units, while minimizing the differences within them [54]. Similarly, the use of a fixed distance band with the Gi* statistic, while useful in the urban portions of Peel, may be somewhat less relevant in the rural (northern) portion, again potentially altering the definition of the Data Zones. In other words, it is important to realize that the processes that create population clusters are unlikely to operate at only one geographic scale, but are instead shaped by complex interactions. Consequently, further work may look at the strengths and weaknesses of the proposed methodology.

## Competing interests

The authors declare that they have no competing interests.

## Authors' contributions

AD and CT completed data collection, analysis, and first draft of the paper. KBN contributed conceptual and methodological pieces, and contributed to the write-up. All authors have reviewed and approved the final text.

## References

[B1] Diez-Roux A Investigating neighborhood and area effects on health Am J Public Health 2001 91 11 1783 89 10.2105/AJPH.91.11.1783 11684601 PMC1446876

[B2] Glazier RH Vahabi M Damba C Patychuk D Ardal S Johnson I Woodward G DeBoer DP Brown A Low H Lawrie C Dudgeon L McConnell S Defining needs-based urban health planning areas is feasible and desirable: a population-based approach in Toronto, Ontario Can J Pub Health 2005 96 5 380 4 10.1007/BF03404037 PMC6976060 16238159

[B3] Ross N Tremblay S Graham K Neighbourhood influences on health in Montreal, Canada Soc Sci Med 2004 59 1485 94 10.1016/j.socscimed.2004.01.016 15246176

[B4] Eyles J Health, environmental assessment and population health: tools for a complex process Can J Pub Health 1999 90 1 S31 S34 10.1007/BF03403576 PMC6980106 10686757

[B5] Flowerdew R Manley DJ Sabel CE Neighbourhood effects on health: Does it matter where you draw the boundaries? Soc Sci Med 2008 66 6 1241 55 10.1016/j.socscimed.2007.11.042 18177988

[B6] Luginaah I Jerrett M Elliott S Eyles J Parizeau K Birch S Abernathy T Veenstra G Hutchinson B Giovis C Health profiles of Hamilton: spatial characterization of neighbourhoods for health investigations Geojournal 2001 53 135 147 10.1023/A:1015724619845

[B7] Lebel A Pampalon R Villeneuve PY A multi-perspective approach for defining neighbourhood units in the context of a study on health inequalities in the Quebec City region International Journal of Health Geography 2007 5 6 27 42 10.1186/1476-072X-6-27 PMC1936419 17615065

[B8] Parenteau M Sawada M Kristjansson EA Calhoun M Leclair S Labonté R Runnels V Musiol A Herold S Development of neighbourhoods to measure spatial indicators of health URISA Journal 2008 20 2 43 55

[B9] Flowerdew R Graham E Feng Scottish Neighbourhood Statistics Data Zones Background Information 2004 http://www.scotland.gov.uk/Publications/2004/02/18917/33248 Report to the Scottish Executive. (Accessed March 8, 2011)

[B10] Weden MM Bird CE Escarce JJ Lurie J Neighborhood archetypes for population health research: Is there no place like home? Health Place 2011 17 289 299 10.1016/j.healthplace.2010.11.002 PMC3085046 21168356

[B11] Fortin MJ Drapeau J Delineation of ecological boundaries: Comparison of approaches and significance tests 72 OIKOS 323 32

[B12] Fortin MJ Effects of data types on vegetation boundary delineation Canadian Journal of Forest Research 1997 27 1851 58 10.1139/x97-156

[B13] Jacquez GM Maruca S Fortin MJ From fields to objects: A review of geographic boundary analysis Journal of Geographic Systems 2000 2 221 41 10.1007/PL00011456

[B14] Lu H Carlin BP Bayesian areal wombling for geographical boundary analysis Geog Anal 2005 37 3 265 85 10.1111/j.1538-4632.2005.00624.x

[B15] Evans RG Bare LM Marmor RT Why are some people healthy and others not? 1994 New York: De Gruyer

[B16] Evans R Stoddart G Producing health, consuming health care Soc Sci Med 1990 31 1347 1363 10.1016/0277-9536(90)90074-3 2126895

[B17] Macintyre S Maciver S Sooman A Area, class and health: should we be focusing on places or people? J Soc Policy 1993 22 2 213 234 10.1017/S0047279400019310

[B18] Getis A Ord JK The analysis of spatial association by use of distance statistics Geogr Anal 1992 24 189 206

[B19] Davies W Urban social structure: A multivariate-structural analysis of Cardiff and its region 1983 Cardiff: University of Wales Press

[B20] Statistics Canada 2006 Community profiles: Peel Region 2010 http://www12.statcan.ca/census-recensement/2006/dp-pd/prof/92-591/details/page.cfm?Lang=E&Geo1=CD&Code1=3521&Geo2=PR&Code2=35&Data=Count&SearchText=peel&SearchType=Begins&SearchPR=01&B1=All&Custom=

[B21] Wilkinson RG Unhealthy Societies: The Afflictions of Inequality 1996 London: Routledge

[B22] Dunn JR Housing and inequalities in health: a study of socioeconomic dimensions of housing and self reported health from a survey of Vancouver residents J Epidemiol Commun H 2002 56 671 81 10.1136/jech.56.9.671 PMC1732232 12177083

[B23] Dunn JR Hayes MV Hulchanski JD Hwang SW Potvin L Housing as a socio-economic determinant of health: Findings of a national needs, gaps and opportunities assessment Can J Pub Health 2006 97 Suppl. 3 S11 S15 10.1007/BF03405392 PMC6975863 17357542

[B24] Ellaway A Macintyre S Fairley A Mums on prozac, kids on inhalers: the need for research on the potential for improving health through housing interventions Health Bulletin 2000 58 336 9 12813815

[B25] Bashir SA Home is where the harm is: inadequate housing as a public health crisis Am J Pub Health 2002 92 5 733 38 10.2105/AJPH.92.5.733 PMC3222229 11988437

[B26] Pottie K Ng E Spitzer D Mohammed A Glazier R Language proficiency, gender and self-reported health: an analysis of the first two waves of the longitudinal survey of immigrants to Canada Can J Pub Health 2008 99 6 505 510 10.1007/BF03403786 PMC6975753 19149396

[B27] Woloshin S Schwatz L Katz S Gilbert Welch HL Is language a barrier to the use of preventive services? J Gen Int Med 1997 12 8 472 477 10.1046/j.1525-1497.1997.00085.x PMC1497155 9276652

[B28] Newbold KB The short-term health of Canada's new immigrant arrivals: evidence from LSIC Ethnicity and Health 2009 14 3 1 22 10.1080/13557850802609956 19263262

[B29] Deri C Social networks and health service utilization J Health Econ 2005 24 6 1076 1107 10.1016/j.jhealeco.2005.03.008 16139910

[B30] Newbold KB Health care use and the Canadian immigrant population Int J Health Serv 2009 39 3 545 565 10.2190/HS.39.3.g 19771955

[B31] Woltman K Newbold KB Immigrant women and cervical cancer screening uptake: A multilevel analysis Can J Pub Health 2007 98 6 470 475 10.1007/BF03405441 PMC6975610 19039885

[B32] Blakely AT Lochner K Kawachi I Metropolitan area income inequality and self-rated health - A multi-level study Soc Sci Med 2002 54 65 77 10.1016/S0277-9536(01)00007-7 11820682

[B33] Dunlop S Coyteb PC McIsaac W Socio-economic status and the utilisation of physicians' services: results from the Canadian National Population Health Survey Soc Sci Med 2000 51 1 123 133 10.1016/S0277-9536(99)00424-4 10817475

[B34] Fiscella K Williams DR Health disparities based on socioeconomic inequities: implications for urban health care Acad Med 2004 79 12 1139 47 10.1097/00001888-200412000-00004 15563647

[B35] Ellaway A Macintyre S Does where you live predict health related behaviours? A case study in Glasgow Health Bulletin 1996 54 443 6 8990608

[B36] Ellaway A Macintyre S Kearns A Perceptions of place and health in socially contrasting neighbourhoods Urban Stud 2001 38 2299 316 10.1080/00420980120087171

[B37] Bonham-Carter GF Geographic information systems for geoscientists: Modelling with GIS 1994 New York: Pergamon

[B38] Ellen IG Turner MA Does neighborhood matter? Assessing recent evidence Hous Policy Debate 1997 8 4 833 66

[B39] Kawachi I Berkman LF Kawachi I, and Berkman LF Introduction Neighborhoods and Health 2004 New York: Oxford University Press 1 19

[B40] Martin DG Enacting neighborhood Urban Geogr 2003 24 5 361 85 10.2747/0272-3638.24.5.361

[B41] Langlois A Kitchen P Identifying and measuring dimensions of urban deprivation in Montreal: an analysis of the 1996 census data Urban Stud 2001 38 1 119 139 10.1080/00420980020014848

[B42] Jolliffe IT Principal Component Analysis 1986 New York: Springer-Verlag

[B43] Norman G Streiner D PDQ Statistics 2003 Toronto: McGraw-Hill

[B44] Primpas I Tsirtsis G Karydis M Kokkoris GD Principal component analysis: development of a multivariate index for assessing eutrophication according to the European water framework directive Ecol Ind 2010 10 2 178 183 10.1016/j.ecolind.2009.04.007

[B45] DMTI Spatial Inc Neighborhood and Community Boundaries 2009 [Computer File]. *v2009.3*.

[B46] DMTI Spatial Inc "DMTI Spatial Releases Neighbourhood and Community Boundaries" 2008 http://www.dmtispatial.com

[B47] Pickett KE Pearl M Multilevel analyses of neighbourhood socioeconomic context and health outcomes: a critical review J Epidemiol Community Health 2001 55 2 111 122 10.1136/jech.55.2.111 11154250 PMC1731829

[B48] Hoxby CM Does competition among public schools benefit students and taxpayers? Am Econ Rev 2000 90 5 1209 38 10.1257/aer.90.5.1209

[B49] Noonan DS Neighbors, barriers and urban environments: are things "different on the other side of the tracks"? Urban Stud 2005 42 10 1817 35 10.1080/00420980500231720

[B50] DMTI Spatial Inc Canmap Route Logistics 2008 [Computer File]. *v2008.3*.

[B51] DMTI Spatial Inc Satellite StreetView [Computer File] 2002 DMTI Spatial Inc

[B52] Townsend P Phillimore P Beatty A Health and deprivation: inequality and the North 1988 (Croom Helm)

[B53] Jelinski DE Wu J The modifiable areal unit problem and implications for landscape ecology Landscape Ecol 1996 11 3 129 140 10.1007/BF02447512

[B54] Openshaw S The Modifiable Areal Unit Problem 1984 Norwich: Geo Books

[B55] Monmonier MS Maximum-difference barriers: an alternative numerical regionalization method Geog Anal 1973 3 245 261

